# Retrospective evaluation of perioperative and short term clinical outcomes in appendicular long bone skeleton fractures repaired via the string of pearls (SOP) locking plate system

**DOI:** 10.1186/s12917-018-1707-6

**Published:** 2018-12-05

**Authors:** Matthew R. Field, Ryan Butler, Robert W. Wills, Wilburn M. Maxwell

**Affiliations:** 1 Arkansas Veterinary Emergency & Specialists, 11619 Pleasant Ridge Road, Little Rock, AR 72212 USA; 2 Mississippi State University, College of Veterinary Medicine, 240 Wise Center Drive, Starkville, MS 39762 USA; 3 Memphis Veterinary Specialists, 555 Trinity Creek Cove, Cordova, TN 38018 USA

**Keywords:** Locking plate, SOP plate, String of pearls, Appendicular fracture

## Abstract

**Background:**

Internal plate fixation and, more recently, locking plate fixation are commonly used in the repair of fractures in small animal surgery. This retrospective study reviewed the use of the String of Pearls locking plate system in the fixation/repair of appendicular long bone skeleton fractures in 31 small animal veterinary patients (33 fractures).

**Results:**

Major complications necessitating revision surgery occurred in 7/33 (21%), with implant failure as the inciting cause in all cases. Variables corresponding to an unsuccessful outcome were evaluated, and a correlation was found with plates placed in a bridging manner (placed without rigid anatomic reconstruction, *p* = 0.02) and length of follow-up (*p* = 0.01).

**Conclusions:**

The SOP plating system can be used in the repair of appendicular longbone skeletal fractures, however, the authors propose that adjunct fixation, such as intramedullary pin, double plating, or external coaptation would likely improve results and should be considered imperative in cases in which anatomic reconstruction is either not desirable or achievable.

**Electronic supplementary material:**

The online version of this article (10.1186/s12917-018-1707-6) contains supplementary material, which is available to authorized users.

## Background

Locking plates were first introduced in human surgery in the late nineteenth century. However, they did not gain widespread acceptance and use until Danis’ work along with Arbeitsgemeinschaft fur Osteosynthesefragen (AO) in the 1960s [1]. Locking plate systems, while traditionally limiting screw insertion angles, offer several advantages that make them attractive alternatives to conventional plating systems. Locking plates act as “internal fixators,” wherein the construct is created by screws locking to the plate and, thus, do not rely on plate-bone friction for stability. This is in contrast with conventional plating techniques in which plates are essentially compressed onto the bone by conventional screws in a lag fashion. Since locking plates do not rely on friction from compression for construct stability, they do not need to be contoured precisely to fit the bone. There are multiple advantages to this: shorter surgical time, reduced bone fragment handling, and, thus, easier maintenance of biologic osteosynthesis if desired [2–5]. Periosteal blood supply is preserved because of the lack of reliance on friction generated between bone and implant with conventional plating, and fractures can be stabilized more easily without interfering with biologic osteosynthesis [2–7]. Locking screws engaging with the plate are angle-stable and resist pullout when compared with non-locking screws [2, 8, 9]. When blood supply is preserved, faster healing times and lower risk of infection may result [2, 8]. Inserting screws in a monocortical fashion may further help to preserve blood supply at the fracture site and also allow the use of orthogonally placed plates [7, 10]. Monocortical screw placement, however, comes at the cost of increased construct compliance [11].

**Fig. 1:**
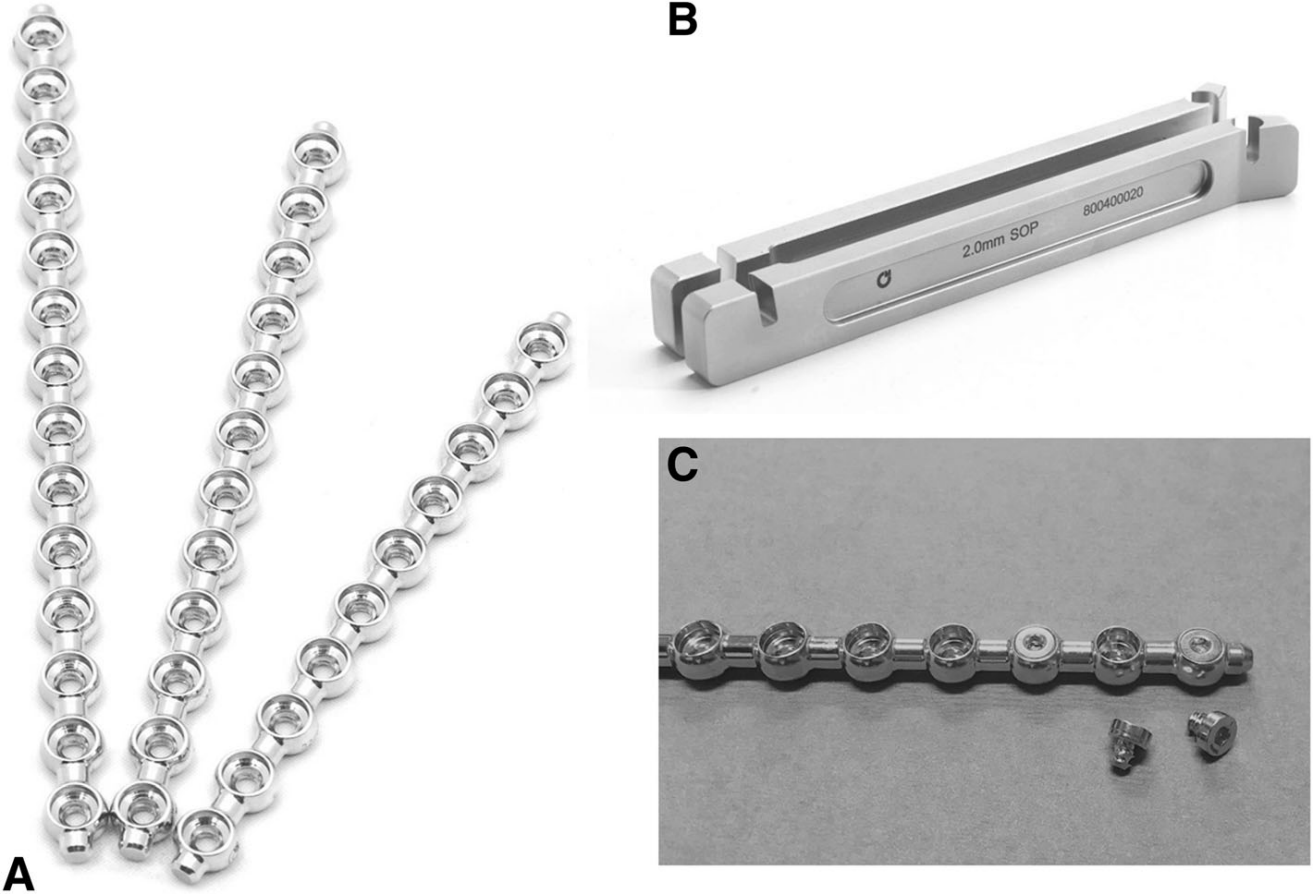
**a** Photo of SOP plate, displaying node and internode areas (**b**) Specialized bending irons for SOP plate, allowing precision bending in multiple planes [**c**] SOP plate with associated bending tees (available for only 2.7 mm and 3.5 mm plates). **a** and **b** reprinted with permission of copyright holder

The SOP locking plate system (Orthomed, UK), a veterinary-specific locking plate, is composed of larger pearls (nodes) for screw insertion separated by internodes, such that the plate vaguely resembles the appearance of pearls on a string. (Figure 1) Plates are made of 316 L stainless steel and accept regular cortical bone screws, either self-tapping or non-self-tapping. Plate sizes are available in 2.0, 2.7, or 3.5 mm. Utilizing standard cortical bone screws is an advantage of the SOP system, as there is no extra inventory to keep on hand in the form of screws. The inside of the pearl portion of the plate is contoured in the shape of a ridge, which allows press-fit of the screw head into the plate. This helps to prevent screw loosening during cyclic loading of the plate [12].

Special plate bending irons are available that interface with the rounded nodes and allow the plates to be contoured in six degrees of freedom: medial to lateral bending, cranial to caudal bending, and torsion. When properly performed, contouring results in bending at the internode site, which helps to preserve the locking function of the pearl portion of the plate [12]. Temporary tee inserts are available for the 2.7 and 3.5 plates for use during contouring to further help preserve the locking function of the plate.

Previous research has shown the SOP plate to be stronger than the LC-DCP plate of comparable size when bending forces are applied [13–16]. SOP plates have also been found to have significantly higher perpendicular load at failure and were found to be stiffer than LC-DCP plates [17, 18]. In the nearly decade that it has been in use, we are only aware of ex vivo, in vitro, and case report studies on the use of SOP plates published in the scientific literature [11, 13–17, 19–31]. The purpose of the present study was to retrospectively evaluate the SOP system in a clinical setting when applied to appendicular skeletal fractures. We hypothesized that the SOP system would prove to be an adequate fixation method when addressing fractures involving the appendicular skeleton in small animal surgery.

## Results

Complete data with follow-up ≥6 weeks were available for 31 patients (30 dogs, 1 cat, 35 SOP applications) consecutively treated for long bone fractures with SOP plate.

### Signalment

Seventeen breeds of dogs (mean weight: 22.2 kg; range 4.5–50.2 kg) and one cat (domestic shorthair) were represented in the present study. Common dog breeds represented included mixed breed (12), Jack Russell Terrier (2), Australian Shepherd (2), and one each of various other dog breeds.

Mean age of treated animals was 3.8 years (range 0.25–16 years). Nine intact males and six neutered males were represented, along with three intact females and thirteen spayed females. Mean follow-up time was 10.8 weeks (range 6.0–34).

Of 31 patients, all received non-steroidal anti-inflammatory drugs except for the feline patient.

### Surgical results and complications

Thirty-one patients were treated for 33 appendicular longbone skeleton fractures. Fractures included femur (19), tibia (4), radius (4), ulna (2), and humerus (4). All patients’ fractures were clinically healed at the end of the follow-up period. The two additional plate applications were orthogonally placed plates.

Fractures encountered included 13 “simple” fractures (classified as spiral, transverse, or oblique), eight “simple” comminuted fractures (defined as a comminuted fracture with four or fewer fragments), and 12 “complex” comminuted fractures (defined as a comminuted fracture with greater than four fragments).

Ten 2.0 mm plates were used in ten patients (median patient weight: 9.8 kg; mean: 16.1 kg; range 4.5 to 50.2 kg). In the largest patient, the 2.0 mm plate was placed as an adjunct fixation to a 3.5 mm SOP plate. Eleven 2.7 mm plates were used in nine patients (mean patient weight: 20.2 kg; range 8.3 to 30 kg). Fourteen 3.5 mm plates were used in 14 patients (mean patient weight: 30.8 kg; range 18.8 to 50.2 kg).

Adjunctive fixation was applied in 19/33 fractures. Sixteen of the 19 utilized cerclage wire, while two others incorporated an intramedullary pin as part of fixation, and one was and augmentation to a fissure fracture in a total hip revision. In one patient (a revision from a previous interlocking nail nonunion), the nail portion of the previous fixation was retained as an intramedullary pin following removal of the interlocking bolts. Two other cases with adjunctive fixation were large patients in which two orthogonally placed plates were used for fixation. All fractures of long bones occurred in the diaphyseal portion of the bone except one which occurred in the metaphysis of the femur. Twenty-three out of thirty-three fractures were repaired with anatomic reconstruction of all cortices and load sharing between the bone and implants (“neutralization”). The remaining ten were repaired without anatomic reconstruction (“bridging”).

**Fig. 2:**
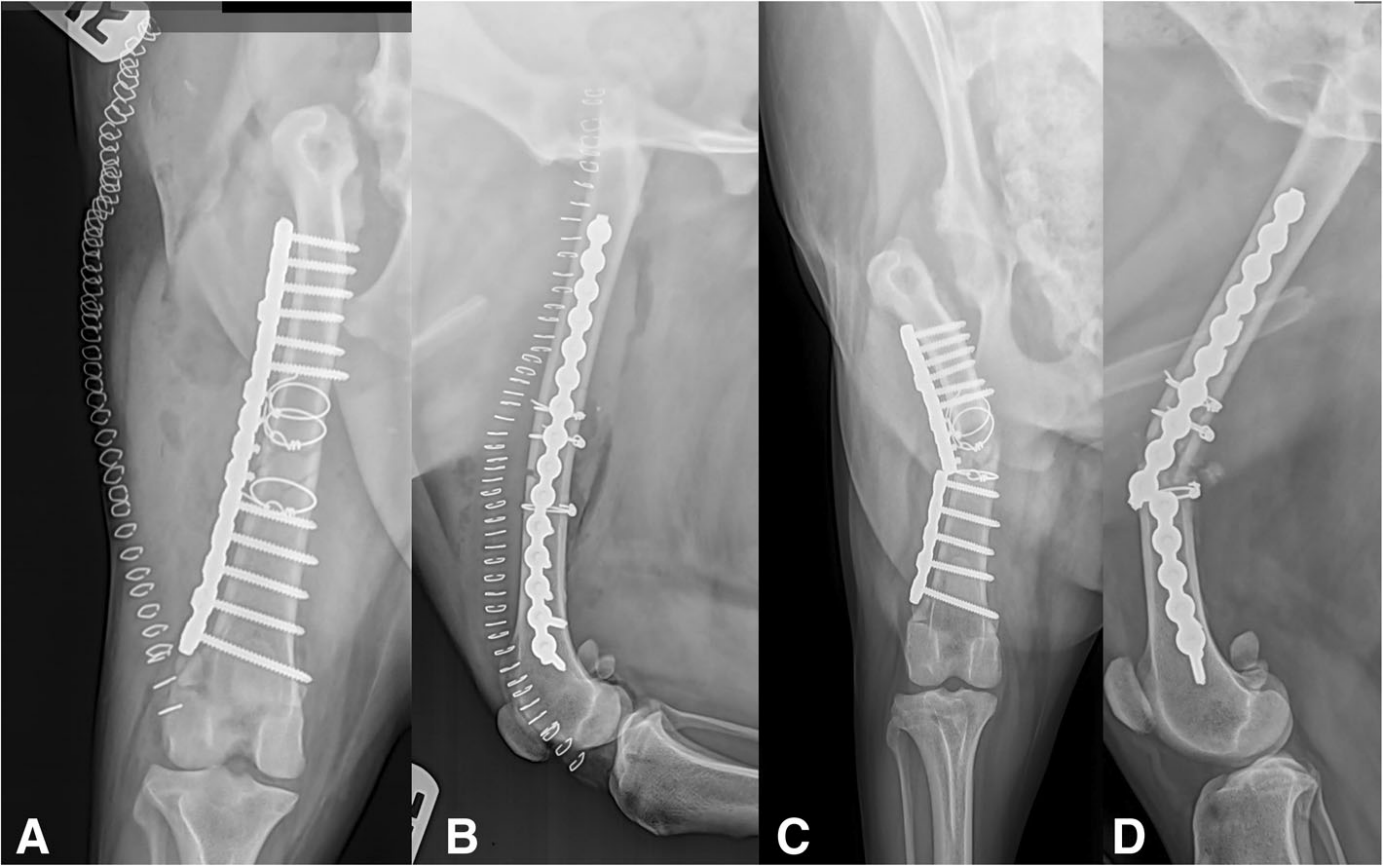
Lateral and craniocaudal immediate postoperative views [**a**] & [**b**] of one of the major complication patients. **c** and **d** illustrate a plate fracture at one of the internode spaces. This patient was one of the “Bridging” patients and likely would have benefited from additional placement of an IM pin

Seven of the 33 fractures (21%) sustained major complications, necessitating revision surgery with alternative fixation (one screw break with non-union, and six broken plates [two of which also had broken screws]). (Figure 2) Of the seven patients with implant failure, five were repaired in a “bridging” fashion. The remaining two were repaired with anatomic reconstruction and load-sharing.

One minor complication was noted in a patient who exhibited post-operative regurgitation which resolved with medical management. With the exception of one of the patients in which the plate broke, all patients were alive at final recheck, with minimal lameness noted. The deceased patient arrested during amputation of the fractured limb at another facility.

### Statistical analysis

After analyzing all variables with the aforementioned methods, the only variables which were statistically associated with higher rate of complication were bridging versus neutralization (*p* = 0.02) and length of followup time (*p* = 0.01). Fractures with longer followup time longer than 10 weeks were 1.5 times more likely to have an unsuccessful outcome. Fractures repaired in a “bridging” manner were 9.12 times more likely to have an unsuccessful outcome compared with those repaired in a “neutralization” manner.

## Discussion

**Fig. 3:**
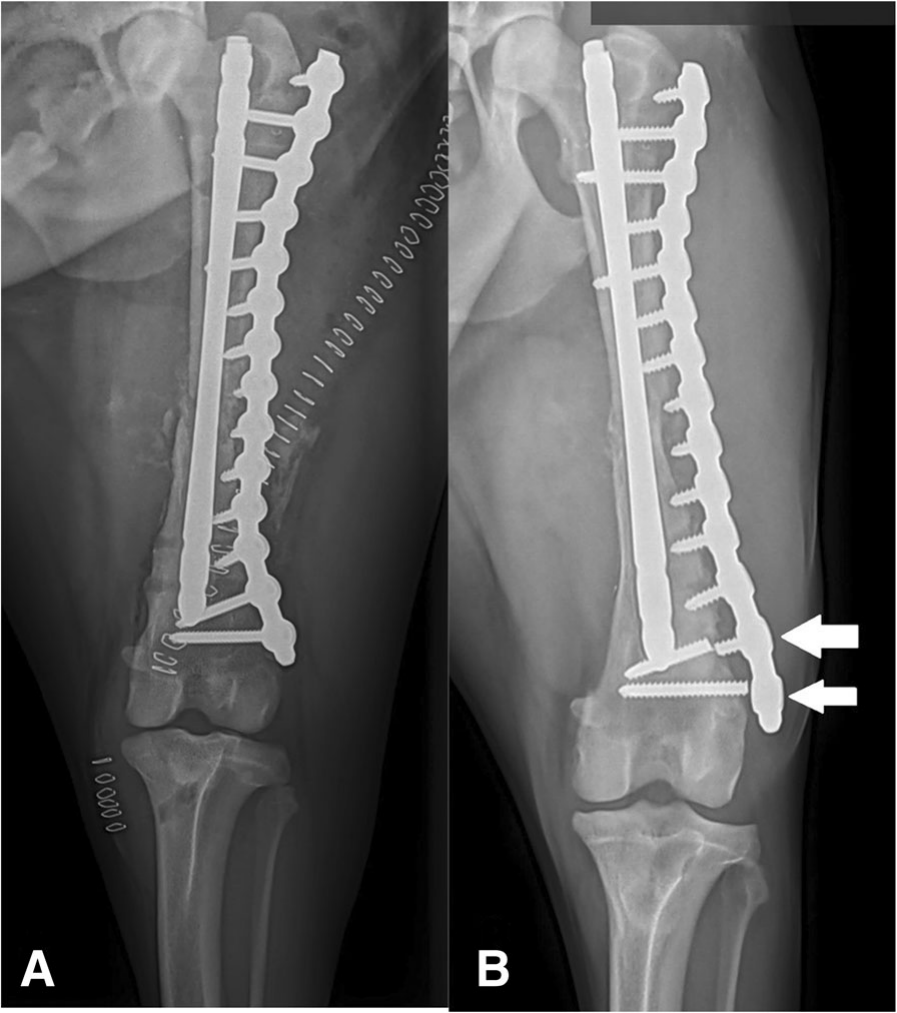
Immediate postoperative radiograph [**a**] of a patient who had revision of a failed interlocking nail repair of a femur fracture. The repair was augmented with autogenous cancellous graft harvested from the proximal tibia and synthetic bone graft substitute. Screw breakage [**b**] (arrows) was noted in this patient six weeks postoperatively

The purpose of our study was to report on the clinical application and outcome associated with SOP plate fixation of appendicular long bone fractures in small animals. The most common major complication reported in the present study was breakage of screws and/or plates. Broken screws were encountered near the plate/screw or the screw/bone interface. (Figure 3) We hypothesized that this was due to a stress riser effect at the SOP plate-screw shaft or screw shaft-bone interface, as has been hypothesized for screw breakage in previous studies [1, 32]. During plate application, stress risers are created at areas where there is a transition from an area with a relatively high area moment of inertia to an area with a lower area moment of inertia (AMI) [33]. AMI is a mechanical concept related to the bending strength of a construct. For solid objects, generally speaking, a larger AMI value means increased resistance to bending forces. In a standard LC-DCP plate, the transition of AMI points occurs at the screw-hole portion of the plate because this is the area with the lowest AMI, and screws are essentially inserted in lag fashion, and thus are not considered a part of the construct. With the SOP system, since the entire construct behaves as one unit, this happens at the transition point of the node (high AMI) and the screw shaft (lower AMI), thus leading to a stress riser effect at the proximal screw shaft. This, in turn, can predispose to breakage of the screw shaft. Additional stress risers exist at the screw-bone interface and at the internode portion of the plate, potentially accounting for breakage of plates at those locations.

It is worth mentioning that locking plates operate on the principle of angular stability, and all locking plates are technically inserted in “bridging” fashion unless other fixation is utilized to obtain significant load-sharing between the bone and the implants [33]. When significant load-sharing does not exist, even in a relatively simple fracture (such as a mid-diaphyseal oblique pattern), compliance of the construct can lead to excessive interfragmentary strain and cyclic loading, which would then result in delayed healing or non-union of fractures, placing more stress on the implant and thus making it more likely to fail. Load sharing between the implant and the bone can be difficult to assess intraoperatively in some cases and may be incorrectly judged. This also is acknowledged as a potential failure point in the patients examined in our study.

Other locking systems take advantage of the previously-mentioned AMI concept and use a screw with a larger screw core diameter [34], thus creating a higher resistance to bending forces on the screw and lessening the likelihood of screw failure. Although this improves the mechanical function of the construct, the additional screw inventory and need for varying drill bits and potentially taps increases the cost associated with the procedure and may increase surgical time. A major cost-saving advantage of the SOP system, as previously noted, is the ability to use standard cortical screws. However, the smaller core diameter of these screws may account for the higher incidence of construct failure (21%) seen in the present study when compared to studies evaluating other locking plate systems (0–9%) [34–37]. Additionally, if the SOP plating system has an unacceptably high failure rate, any cost savings gained in inventory may be negated by costs of additional surgery.

AO suggests differing guidelines in the treatment of non-reconstructable comminuted fractures in human trauma [38]. When a fracture is deemed “complicated,” plate span width and plate screw density recommendations are different than a “simple” fracture. Recommendations in human medicine are that the plate length should be two to three times higher than the overall fracture length in comminuted fractures versus the eight to ten times higher recommended in simple fractures. Data from human studies suggests that plate-screw density should be below 0.5. It is unknown if these recommendations can be routinely applied to veterinary medicine, as our ability to control a patient’s post-operative activity is understandably limited compared with human medicine. In the current study, neither plate-screw density nor plate span length comparisons reached statistical significance.

Other veterinary locking plate systems have had different results compared to our current findings with the SOP plating system. A recent study evaluating the use of a titanium locking plate did not find a major complication rate as high as the current study [34]. In that study, a 5.5% major complication rate was noted, contrasting with our complication rate of 21%. Other studies investigating the use of the locking compression plate on fractures have found major complication rates necessitating revision in 0–9% of fractures [35–37]. In these studies, the predominant modes of failure were screw pullout, bending of plates, and breakage of plates.

Failure of locking plates in human surgery has been noted from several standpoints. Physiological loads beyond plate-design parameters, screw disengagement due to cross-threading, insufficient screw torque, and excessive cyclical loading have all been noted as causes of failure [1]. The authors believe that the modes of failure for the current study were likely the result of cyclical loading or physiologic overloading due to patient excessive activity, although the other causes cannot be completely ruled out. In future studies, failed implants might be examined with scanning electron microscopy to evaluate for modes of failure, as has been done in human and veterinary medicine [39, 40].

Previous studies have indicated that the SOP plate is at least equivalent or stronger in bending, torsion, and when perpendicular loads are applied than a comparable conventional LC-DCP plate [13–16, 18]. However, it is prudent to keep in mind that kinetic variables measured ex-vivo (bending or torsional strength, etc.) only approximate forces that the implants will see during actual load bearing. Failure of surgical implants is more often a consequence of cyclic loading over time rather than a single large physiological force application [33]. In reality, our patients likely see a variety of force magnitudes on fracture repairs; none of the patients in this study reported any catastrophic events that could be associated with a large force application. Examples of such a force application might include slipping on the floor, being involved in motor vehicle trauma, or falling from a height.

Statistical analysis from our data indicated that follow-up times were longer in patients in which an unsuccessful outcome was noted. This is a somewhat expected outcome, and likely occurred because our follow-up times were necessarily increased in patients in which delayed healing and/or implant failure were noted and, subsequently, revision surgery was necessary.

Analysis of our data also indicated that fractures which were repaired in a “bridging” manner were greater than nine times as likely to fail as those repaired in a “neutralization” manner. The authors chose this terminology for ease of comparison and classification of fractures. As previously mentioned, all locking plates are technically placed in bridging fashion, though there can be varying levels of load sharing and interfragmentary strain depending on level of anatomic reconstruction and adjunct fixation utilized. Of the seven unsuccessful outcomes noted in the current study, five were in patients whose fractures were repaired in a “bridging” fashion. Three of the five had no adjunct fixation applied. Only four of the adjunct fixation cases utilized implants that would directly provide additional bending strength; the cerclage utilized most often instead relies on bone integrity to reduce strain on the SOP plate.

Both plate-screw density and plate:bone ratio were assessed for complication rates to look for the possibility of inappropriately repaired fractures (at least when compared to human recommendations for fractures repaired in bridging fashion). No correlation was found with regard to unsuccessful outcomes; this is potentially due to a type 2 statistical error.

The use of nonsteroidal anti-inflammatory drugs (NSAIDs) in human patients undergoing orthopedic surgery remains controversial. A recent literature review detected conflicting data on delayed bone healing in human and lab animal patients [37]. Due to the conflicting evidence in human medicine, the authors of this study wanted to include the administration of anti-inflammatory drugs as a potential confounding variable. Due to only one patient in the present study who did not receive NSAIDs, however, no meaningful conclusions could be drawn.

The mean follow-up time in this study classifies it as a perioperative to short-term study, as previously defined by Cook et al. [41] As an investigative study relating to the clinical applications of the SOP plating system for bone healing, it was beyond the scope of the current study to evaluate mid- and long-term outcomes. Further questions to be investigated in a longer-term study would include whether this plating system would have any effect on ambulation, scar tissue formation, or future occurrence of infection or neoplasia.

Potential solutions for the issue of implant failure noted in our study would be to augment fixation with implants that provide increased bending resistance, such as an intramedullary pin or a second, orthogonally placed, plate, particularly in patients with highly comminuted fractures that are unable to be neutralized or repaired anatomically. It has previously been shown that the addition of an intramedullary pin can decrease strain on a bone plate by as much as ten-fold [42]. As such, the addition of an intramedullary pin would greatly increase bending strength and may help prevent cyclical causes of failure. It is also difficult to predict the amount of activity a given patient will have and subsequent forces generated. Because of this, adjunct fixation could also be considered, whether in the form of adjunctive fixation (IM pin, orthogonal plating, etc.) or external coaptation such as splints, or temporary non-weight-bearing bandages (90–90, carpal flexion, etc.). However, the benefit of external coaptation must be weighed against potential complications.

It is possible that the method of fixation used in our study had an impact on fracture healing, and subsequently, implant failure. All SOP applications in this study utilized an open reduction with internal fixation method. The decision to repair the fractures in this manner was mainly dependent on surgeon experience with minimally invasive techniques and a lack of access to minimally invasive equipment. Minimally invasive plate osteosynthesis (MIPO) has gained favor in recent years, as it better preserves blood supply to the fracture site, and it has been shown to maintain viability of some smaller fractures segments [35]. The use of a radiopaque synthetic bone graft means it is possible that some plate applications involved less than ideal neutralization of load-bearing forces; these fractures (such as that shown in Fig. 2) may have been better suited to a “look but do not touch” open fracture stabilization technique and placement of the plate in a bridging fashion to better preserve the fracture blood-supply [33].

There were several limitations to the current study. Because of its retrospective nature and the lack of a comparison cohort, direct comparisons with other plating systems, especially locking systems, cannot be made. Indirect comparisons, therefore, were made with other systems when warranted. There were a limited number of cases in our study, especially considering the many variables that can affect the success of fracture fixation. It is possible with a higher number of patients, other variables associated with failure may reach statistical significance. With the exception of the two patients previously mentioned with two orthogonal plates, the patient in Fig. 3 with an interlocking nail revision, and one additional patient with an IM pin, none of the patients in the current study had adjunct fixation other than cerclage wire applied to the fracture. The manufacturer of the SOP system recommends the use of an intramedullary pin when used for long bone fractures and double plating for larger patients, but ultimately leaves the use of adjunct fixation up to the individual surgeon [12]. Given the ample biomechanical studies indicating the SOP plate stiffness [13–16, 18], we felt that such additional fixation might not be necessary. However, since the introduction of the SOP system, Rutherford et al. have found that adding an IM pin with 32% medullary canal diameter produced comparable construct compliance and angular deformation to a traditional plate-rod construct in a fracture gap model [19]. It is possible that the introduction of an intramedullary pin or other adjunct fixation would have improved construct durability in the current study.

Due to the use of radiopaque bone graft for all of the fractures in the current study, it is possible that the number of fractures repaired using plates in bridging fashion was underestimated. The opacity of the bone graft in some cases limited the assessment of reconstruction of the transcortex and might lead to an overestimation of load sharing between the bone and implant.

Another potential limitation is that we did not use a torque-limiter for screw insertion. The manufacturer of the SOP system recommends tightening screws so that the screw head seats firmly into the spherical component of the SOP plate [12]. This is practical advice, but it could potentially lead to under- or over-tightening of screws, which could fatigue the metal and also predispose to implant failure. We could not rule this out as a potential cause of implant failure.

## Conclusions

Based on the results of the current study, we concluded that while the SOP plating system can be used to treat longbone fractures, precautions should be taken when using the plates in a bridging fashion, and adjunctive fixation that augments bending strength (in the form of intramedullary pin, orthogonal plating, external coaptation, etc.) is strongly recommended. Further in vivo study is recommended to further assess these adjunct fixations, as well as other potential confounding variables, such as plate span ratio or plate screw ratio.

## Methods

### Inclusion criteria

Medical records (May 2013–May 2016) from all consecutive dogs and cats with long bone fractures treated with SOP at Memphis Veterinary Specialists (MVS) were reviewed. Only clinical patients that had radiographic follow-up confirming bone healing after surgery were included in this study. For patients who had rechecks with the referring veterinarian, a copy of recheck radiographs was obtained and evaluated. Either a boarded surgeon or a surgical resident under the direct supervision of a boarded surgeon performed all surgeries. Radiographs were reviewed by a board-certified surgeon to assess post-operative and follow-up progress. For patients whose radiographs were obtained from the referring veterinarian, owners or referring veterinarians were questioned as to the patient’s degree of function. Patients who were not considered clinically and radiographically healed at recheck were instructed to return every four weeks for an additional recheck until the fracture was considered healed.

### Exclusion criteria

Patients with SOP applied for conditions other than fractures of long bones of the appendicular skeleton, those who did not return for follow up, and for whom no recheck data could be obtained (failed to return or deceased) were excluded from this study.

### Data retrieved

Information retrieved from medical record and radiographs included: breed, body weight, age at time of surgery, gender, indication for surgery, plate size, plate length (measured as number of plate holes), manner repaired (defined as either “bridging” or “neutralization”), plate span ratio (ratio between the length of the fracture and the length of the plate [always > 1]), plate:bone ratio (ratio between the length of the bone and the length of the plate [always ≤1]), screw density (the ratio between number of holes in the plate and the number of screws used [always ≤1]), concurrent disease, follow-up time (in weeks), outcome, and complications.

Plates were considered placed in a “neutralization” fashion if, either through the use of adjunct fixation or a simple fracture configuration, anatomic reconstruction was possible such that absolute load sharing was achieved. All other plates applied to fractures in which strict anatomic reconstruction and load sharing could not be achieved, whether or not adjunct fixation was used, were considered placed in a “bridging” fashion.

Outcomes and complications were reported in a manner consistent with previous recommendations in outcome reporting in veterinary orthopedic surgery [41]. For the purpose of statistical analysis for this study, outcomes were assigned as either “Successful” or “Unsuccessful.” Successful outcomes were defined as those fractures that healed without the need for revision (major complication) and no or mild lameness at recheck. All other outcomes were unsuccessful. A detailed case-by-case description is available as Additional file 1.

### Surgical technique

All patients underwent open reduction with internal fixation of all fractures. Attempt at anatomic reconstruction was made for fractures oriented in a transverse, oblique, spiral, or mildly comminuted (≤4 large fracture fragments) pattern. Severely comminuted fractures were repaired by placing the SOP plate in a bridging fashion without attempt at anatomic reconstruction. Plates were contoured and fit as close to the bone surface as reasonably possible prior to application, and a minimum of three screws per fracture segment were inserted. Any remaining empty screw holes were filled with short screws (non-engaging) or bending tees to improve the strength of the overall construct. In some cases (at surgeon’s discretion), adjunctive fixation was used in the form of cerclage wire, intramedullary pin, or double plating. Synthetic bone graft (Velosity Bioactive Synthetic Bone Graft, Securos, UK) was placed at all fracture sites prior to closure.

### Statistical analysis

Variables evaluated for correlation with major complications or revisions included age at the time of surgery, gender, weight at the time of surgery, plate size, use of adjunct fixation, length of plate (measured by number of holes), bridging versus neutralization, bone segment in which the fracture occurred, fracture configuration (transverse, oblique, spiral, mildly comminuted [≤4 fragments], severely comminuted [> 4 fragments]), plate span width, plate:bone ratio, plate:screw density, and followup time in weeks. To determine the association between the occurrence of complications and each explanatory variable, separate univariable logistic regression models were fit with PROC LOGISTIC in SAS for Windows 9.4 (SAS Institute, Inc., Cary, NC). Penalized maximum likelihood estimation was used for the bone model due to quasi-complete separation of data points. Variables with a *p*-value of less than or equal to 0.26 were selected as candidates for a multivariable model. Collinearity was assessed by determining the correlation among quantitative variables using PROC CORR. A manual backward selection process was followed for variable selection, in which after fitting the model, the variable with the greatest *p*-values was removed and the model refit. This was continued until the final model contained only variables with a p-value of less than 0.05. Hosmer and Lemeshow Goodness-of-Fit Test was used to assess the model fit.

## Additional file

Additional file 1: Table with individual cases and data points gathered for each. (PDF 237 kb)

## Abbreviations

AMI: Area Moment of Inertia
AO: Arbeitsgemeinschaft fur Osteosynthesefragen
IM: Intramedullary
LC-DCP: Limited Contact Dynamic Compression Plate
MIPO: Minimally Invasive Plate Osteosynthesis
MVS: Memphis Veterinary Specialists
SOP: String of Pearls
UK: United Kingdom
