# Supplementary material for: Retrospective evaluation of perioperative and short term clinical outcomes in appendicular long bone skeleton fractures repaired via the string of pearls (SOP) locking plate system
Source: BMC Vet Res. 2018 Dec 5;14:386. doi: 10.1186/s12917-018-1707-6 (PMC6282292; doi:10.1186/s12917-018-1707-6)
Supplement: Supplementary file 1 — Table with individual cases and data points gathered for each. (PDF 237 kb) [file 12917_2018_1707_MOESM1_ESM.pdf]

Patient	Plate	Age (Yrs)	Sex	Breed	Weight	Bone	PlateSize	Holes	FollowUp	AdjunctFix	Bridge Neutral	Bone Region	Fx Config	PlateSpanW	PlateBoneR	PlateScrewD	Complication
1	1	1.0	MN	Jack Russell	8.3	Femur	2.7	11	6.5	None	N	D	T	37.5	0.72115385	0.90909091	NO
2	2	0.66	M	Lab Mix	25.2	Radius	3.5	8	8.1	None	N	D	C: <=4	3	0.60810811	1	NO
3	3	0.4	M	Pit Mix	11.3	Femur	2.7	10	6.2	None	B	D	C: >4	7.66666667	0.67647059	1	YES
4	4	2.0	FS	Great Dane	50.2	Femur	2.0	16	10.1	IM, C, DP	B	D	C: >4	2.25	0.47368421	0.4375	NO
4	5	2.0	FS	Great Dane	50.2	Femur	3.5	12	10.1	IM, C, DP	B	D	C: >4	2.75	0.57894737	0.58333333	NO
5	6	1.0	M	Lab Mix	30.2	Femur	3.5	12	13.3	C	N	D	C: <=4	4	0.8	0.75	NO
6	7	3.0	M	Border Collie	32.2	Radius	3.5	12	11.4	None	B	D	T	12.2	0.84722222	1	YES
7	8	2.5	FS	Mastiff	34.0	Tibia	3.5	15	10.2	C	B	D	C: >4	1.82857143	0.98461538	0.53333333	NO
8	9	9.1	FS	Golden Retriever	32.6	Femur	3.5	14	12.0	C	N	D	C: <=4	2.53846154	0.78571429	0.85714286	NO
9	10	1.75	M	Jack Russell	7.4	Femur	2.0	9	10.2	C	N	D	O: Long	3.6	0.83076923	0.88888889	NO
10	11	12.0	FS	Keeshond	16.2	Femur	2.7	13	32.4	C	N	D	C: <=4	2.5	0.67307692	0.69230769	YES
6	12	3.25	M	Border Collie	34.0	Ulna	2.0	7	11.4	None	N	D	T	27	0.28125	0.85714286	NO
11	13	2	M	Lab Mix	34.7	Tibia	3.5	13	6.4	None	B	D	C: >4	8.66666667	0.8125	1	NO
12	14	6.9	FS	Chi Mix	7.7	Ulna	2.0	6	15.5	None	N	M	T	13.5	0.47368421	0.83333333	NO
13	15	3.33	FS	Aust. Shep. X	22.2	Radius	2.7	13	10.2	None	N	D	T	16.6666667	0.72463768	1	NO
13	16	3.33	FS	Aust. Shep. X	22.2	Radius	2.7	13	10.2	None	N	D	T	17.3333333	0.7027027	1	NO
14	17	1.0	MN	German Shepherd	25.7	Humerus	3.5	10	6.1	C	N	D	C: <=4	3.93333333	0.67045455	0.8	NO
15	18	16	FS	DSH	4.5	Femur	2.0	11	6.1	C	N	D	O: Long	5.44444444	0.80327869	0.72727273	NO
16	19	0.25	M	Pit Mix	9.0	Femur	2.0	9	6.6	None	B	D	C: >4	4.5	0.58441558	0.77777778	NO
17	20	3.3	MN	Pit Mix	32.0	Femur	3.5	14	19.5	ILN	N	D	T	6.27272727	0.85185185	0.85714286	YES
18	21	1.5	FS	Aust. Shep	21.5	Femur	2.7	16	6.4	C	N	D	C: >4	2.43478261	0.8115942	0.5625	NO
19	22	0.5	F	Chesapeake Bay Retriever	24.5	Femur	3.5	12	11.5	None	N	D	C: <=4	5	0.8	1	NO
20	23	0.5	F	Great Dane Mix	18.8	Femur	3.5	9	6.0	C	N	D	C: <=4	2.94117647	0.68493151	0.66666667	NO
21	24	2.0	F	Labrador Retriever	32.6	Femur	3.5	12	7.3	C	N	D	C: <=4	5.77777778	0.69333333	0.75	NO
22	25	12.6	FS	Shelti	16.6	Tibia	2.0	13	34.4	C, Screw	B	D	C: >4	4.75	0.79166667	0.76923077	YES
23	26	3.0	MN	Catahoula	30.6	Femur	3.5	15	15.6	C	B	D	C: >4	4.33333333	0.7027027	0.73333333	YES
24	27	5.33	M	Aust. Shep	19.0	Tibia	2.7	16	16.4	None	B	D	C: <=4	8.83333333	0.70666667	0.9375	YES
25	28	0.5	M	Pit Mix	14.4	Femur	2.0	16	6.5	C	B	D	C: >4	2	0.9122807	0.6875	NO
26	29	9.0	FS	Boston Terrier	10.7	Humerus	2.0	11	6.0	C	N	D	C: >4	2.01666667	0.82876712	0.54545455	NO
27	30	1.33	FS	Mixed	25.0	Humerus	2.7	11	6.2	IM	N	D	O: Short	9.06666667	0.77714286	1	NO
28	31	1.2	FS	Pit Bull	28.1	Femur	3.5	12	7.3	None	N	D	C: >4	3.57142857	0.8	0.83333333	NO
29	32	0.75	FS	Chi Mix	6.0	Femur	2.0	8	7.4	None	N	D	O: Short	9.875	0.69911504	1	NO
30	33	10.3	MN	Sheepdog	30.0	Femur	2.7	11	9.1	THR	N	D	O: Long	2	0.4	0.81818182	NO
30	34	10.3	MN	Sheepdog	30.0	Femur	2.7	20	9.1	THR	N	D	O: Long	3.5	0.7	0.9	NO
31	35	2.7	MN	English Bulldog	16.2	Humerus	2.7	10	5.0	C	N	D	O: Long	2.25	0.68275862	0.8	NO
